# The alveolate translation initiation factor 4E family reveals a custom toolkit for translational control in core dinoflagellates

**DOI:** 10.1186/s12862-015-0301-9

**Published:** 2015-02-10

**Authors:** Grant D Jones, Ernest P Williams, Allen R Place, Rosemary Jagus, Tsvetan R Bachvaroff

**Affiliations:** Institute of Marine and Environmental Technology, University of Maryland Center for Environmental Science, Baltimore, USA; University of Maryland, Baltimore, Graduate School, Baltimore, USA

**Keywords:** Dinoflagellates, Translation initiation, eIF4E, Phylogeny, Alveolate, Heterokont

## Abstract

**Background:**

Dinoflagellates are eukaryotes with unusual cell biology and appear to rely on translational rather than transcriptional control of gene expression. The eukaryotic translation initiation factor 4E (eIF4E) plays an important role in regulating gene expression because eIF4E binding to the mRNA cap is a control point for translation. eIF4E is part of an extended, eukaryote-specific family with different members having specific functions, based on studies of model organisms. Dinoflagellate eIF4E diversity could provide a mechanism for dinoflagellates to regulate gene expression in a post-transcriptional manner. Accordingly, eIF4E family members from eleven core dinoflagellate transcriptomes were surveyed to determine the diversity and phylogeny of the eIF4E family in dinoflagellates and related lineages including apicomplexans, ciliates and heterokonts.

**Results:**

The survey uncovered eight to fifteen (on average eleven) different eIF4E family members in each core dinoflagellate species. The eIF4E family members from heterokonts and dinoflagellates segregated into three clades, suggesting at least three eIF4E cognates were present in their common ancestor. However, these three clades are distinct from the three previously described eIF4E classes, reflecting diverse approaches to a central eukaryotic function. Heterokonts contain four clades, ciliates two and apicomplexans only a single recognizable eIF4E clade. In the core dinoflagellates, the three clades were further divided into nine sub-clades based on the phylogenetic analysis and species representation. Six of the sub-clades included at least one member from all eleven core dinoflagellate species, suggesting duplication in their shared ancestor. Conservation within sub-clades varied, suggesting different selection pressures.

**Conclusions:**

Phylogenetic analysis of eIF4E in core dinoflagellates revealed complex layering of duplication and conservation when compared to other eukaryotes. Our results suggest that the diverse eIF4E family in core dinoflagellates may provide a toolkit to enable selective translation as a strategy for controlling gene expression in these enigmatic eukaryotes.

**Electronic supplementary material:**

The online version of this article (doi:10.1186/s12862-015-0301-9) contains supplementary material, which is available to authorized users.

## Background

Dinoflagellates are unusual eukaryotic organisms well known for bloom formation in coastal waters [1], making toxins that bioaccumulate in the food chain [2], producing bioluminescence [3], and as coral symbionts. Dinoflagellate nuclei differ from those of other eukaryotes, exhibiting large birefringent chromosomes that lack typical eukaryotic nucleosomal DNA packaging. These chromosomes remain condensed during interphase and in many cases throughout the entire cell cycle [4]. Dinoflagellate genomes can also be very large, up to hundreds of picograms of DNA per nucleus, making comprehensive genomic sequencing very difficult [5-7]. In addition, many dinoflagellate genes are duplicated with between tens to thousands of copies found for genes such as the peridinin chlorophyll protein, proliferating cell nuclear antigen, RuBisCO, light harvesting protein, luciferin binding protein, and actin [8-18]. The unusual features of the dinoflagellate nucleus are also reflected by atypical transcription [19].

In general, dinoflagellate gene expression appears to be controlled at the level of translation rather than transcription. For example, bioluminescence in dinoflagellates is strictly diurnally regulated, with little bioluminescence during the day and a peak during the night. Luciferin binding protein is made primarily at night, but transcript levels for the protein remained unchanged over a diel cycle [20]. This example of translational regulation has been validated and expanded by numerous studies of heat shock, nutrient stress, salinity and other conditions using microarrays and high-throughput sequencing [21-26]. These studies have demonstrated a nearly constant transcriptome profile during stress with only small changes in transcript levels [27].

Given that dinoflagellates show little transcriptional control of gene expression, we looked for innovations in translation initiation unique to dinoflagellates that might enable specific translational control. Furthermore, dinoflagellate mRNA cap structures derive from the *trans*-splicing of a 22-nucleotide fragment from an independently transcribed molecule, the spliced leader RNA [28,29], which has a unique cap structure [30]. In view of this, we have placed emphasis on a key step in mRNA recruitment, the binding of eukaryotic translation initiation factor 4E (eIF4E) to the modified base at the 5′ terminus of the mature mRNA (mRNA cap).

**Figure 1:**
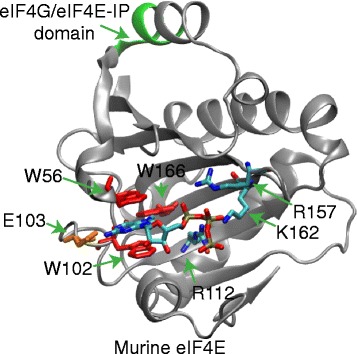
**The structure of murine translation initiation factor 4E (eIF4E-1) with mRNA cap showing key binding residues.** The crystal structure of Class I murine eIF4E, [PDB:1L8B], was used to show conserved binding domains. Residues W56, W102, and W166 are highlighted in red, as well as E103, which is highlighted in orange, directly interact with the methyl-guanosine moiety. Residues R112, R157 and K162, which are highlighted, contribute charged interactions with the phosphate bridge that links the m^7^GTP to the rest of the mRNA chain. A key conserved domain that interacts with eIF4G or eIF4E-interacting proteins is colored green on alpha helix-1.

eIF4E is defined by the cupped hand structure within which the mRNA cap is bound, as exemplified by the mouse eIF4E [PDB:1L8B], a prototypical metazoan Class I cap binding translation initiation factor (Figure 1) [31,32]. This novel fold is characteristic of the eIF4E family. The mRNA cap-binding region is found within a core of 160 to 170 amino acids containing eight aromatic residues with conserved spacing [33]. The secondary structure consists of six beta sheets and three major alpha helices [31,32]. The beta sheets line the binding pocket, and recognition of the 7-methylguanosine moiety is mediated by base sandwich-stacking between conserved residues W56 and W102 as well as with E103. In addition, W166 interacts with the methyl group on the modified base of the mRNA cap. Furthermore, the triphosphate of the cap forms salt bridges with R112, R157 and K162 [31,32]. The alpha helices form the exterior, solvent accessible side of the protein. Alpha helix one, containing the recognition motif of S/TVxxW interacts with eukaryotic translation initiation factor 4G (eIF4G) and eIF4E-interacting proteins [34].

eIF4E is part of an extended gene family found exclusively in eukaryotes. Throughout the eukaryotic domain, a series of eIF4E gene duplications has led to the generation of a family of proteins with multiple structural classes and in some cases subclasses within a given organism [30,33,35]. Although the family is named for the translation initiation factor, not all members of the gene family function as translation factors [36,37]. This family is comprised of proteins predicted to have the novel fold. One or more eIF4E cognates bind the 5′- cap structure of mRNA, a key step in mRNA recruitment to the ribosome. Other eIF4E family members interact with specific mRNAs or proteins to regulate translation of those mRNAs rather than participate in global translation initiation [38-41]. Additional roles undertaken by eIF4E family members include nuclear transport, sequestration and turnover of mRNA [34-36]. Furthermore, some family members regulate translation of specific mRNAs rather than participate in global translation initiation. The eIF4E family can be seen as a toolkit for post-transcriptional regulation of gene expression [42].

It has been noted that the nomenclature for eIF4E family members has evolved with confusion [43]. Eukaryotes have been reported to have at least one and up to eight members of the eIF4E family [33,35]. One proposed classification has divided the different members of the eIF4E family from multicellular organisms into structural classes; Class I, Class II, and Class III [33]. Phylogenies of the eIF4E family are poorly resolved, but support monophyly of metazoan Classes I-III. Thus, while the structural classes define homologous, monophyletic clades within metazoans, across eukaryotes the evolutionary history of the family remains obscure [30].

**Figure 2:**
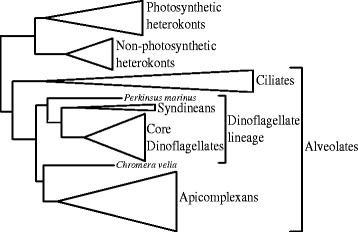
**The organismal phylogeny of the species used for the eIF4E geneology.** The schematic phylogeny is based on the tree from a concatenated matrix of 75 ribosomal protein genes with only well supported clades shown (Adapted from [44]). The collapsed triangular clades correspond in height to the number of species and the width is proportional to branch length.

Here we describe the phylogeny of the eIF4E family in dinoflagellates and a nested series of outgroups. The ‘core dinoflagellates’ have the expanded nuclear genomes described above and encompass the common bloom forming species [44,45]. From the core dinoflagellates, the parasitic syndineans from the genus *Amoebophrya* and *Perkinsus marinus* represent the primary outgroups; which, taken together, form the broadly defined dinoflagellate lineage [46-48]. In turn, the dinoflagellate lineage, apicomplexans, and ciliates form a large clade called the alveolates, with well-established organismal phylogenetic relationships (Figure 2) [44,49]. The next closest outgroup with good sequence representation would be the heterokonts (stramenopiles) [50,51]. Comparing the genealogy of the eIF4E family with the organismal relationships helps determine the relative timing and extent of duplications. The expanding transcriptome datasets for dinoflagellates provide sufficient taxon sampling for this analysis. The goal is to see if eIF4E gene duplications unique to core dinoflagellates can be related to novel functions in translational control of gene expression and to create a nomenclature for the dinoflagellate eIF4E family that reflects evolutionary history.

## Results

### The gene census and major clades for the heterokont and alveolate eIF4E family

The core dinoflagellate eIF4E family contains between eight and fifteen members per species with a total of 126 from eleven species (Additional file 1). In contrast, the members of the dinoflagellate lineage outside of the core dinoflagellates, *Perkinsus marinus* and *Amoebophrya* contain seven and three members per species, respectively. Of the sequences available for the other alveolate groups investigated (15 apicomplexans, 6 ciliates); apicomplexans have one or two family members per species, and ciliates have one to four. All of the ciliate sequences within eIF4E-1 and several within eIF4E-2 showed biased amino acid composition when compared with the remaining sequences, and ciliates consistently formed the longest branches in the phylogeny (Additional file 2) [52]. The outgroup heterokonts (14 species) contain between one and six eIF4E family members per species.

**Figure 3:**
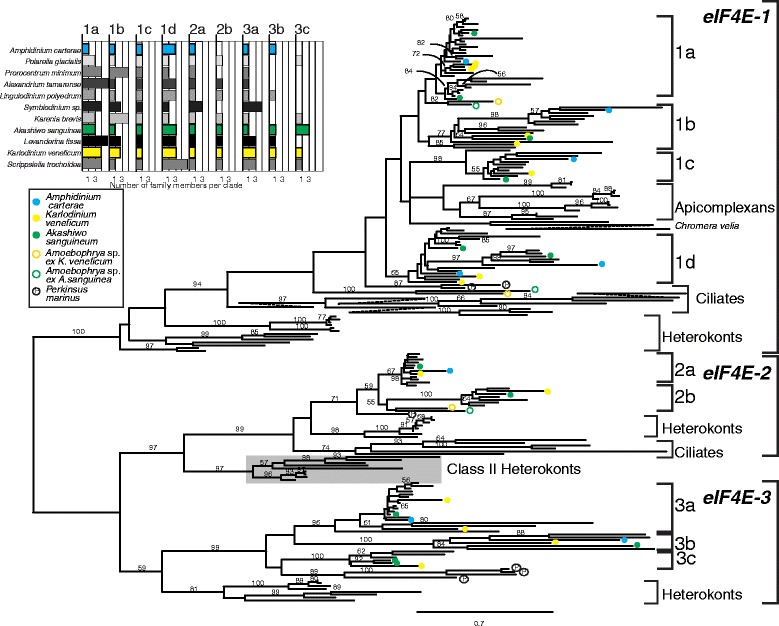
**A maximum likelihood phylogeny of the core eIF4E domain using the Le and Gascuel (LG) amino acid substitution matrix with gamma site to site rates and including representatives of the three major alveolate lineages (ciliates, apicomplexans, and dinoflagellates) and the outgroup heterokonts.** Bootstrap values (1000 replicates) above 55% are shown. The three major clades are highlighted with the right-most brackets. The major clades were defined by the presence of core dinoflagellates and the outgroup heterokonts. The different heterokont, ciliate and apicomplexan clades are labeled, while the nine core dinoflagellate sub-clades are denoted with brackets and the clade names used in the text. The individual species labels have been removed for clarity with three examples of core dinoflagellates represented by colored dots as shown in the key. The distribution of two syndinean dinoflagellates and *Perkinsus marinus* are also shown. The Class II eIF4E found only in heterokonts is shown in a grey background. Long branches for the ciliates in eIF4E-1 and *Chromera velia* were broken and joined with dotted lines to fit onto the page. The inset category plot shows the number of eIF4E family members in each of the nine eIF4E sub-clades across all eleven core dinoflagellates with the same color coding for the three example core dinoflagellate species as on the phylogeny. The tree including genus and species names is available in Additional file 3 in nexus format.

The eIF4E phylogeny contains three major clades (Figure 3, Additional file 3). These clades were defined after tree construction based on representation from both core dinoflagellates and heterokonts, based on the assumption that heterokonts form an outgroup to the alveolates (Figure 2). Here we use letters to designate core dinoflagellate sub-clades, *e.g.* eIF4E-1a. In total, there are nine eIF4E sub-clades in the core dinoflagellates (see Figure 3 inset). All three alveolate lineages surveyed are represented in eIF4E-1, as well as the outgroup heterokonts. The branching pattern within eIF4E-1 roughly corresponds with organismal relationships (Figure 2) [44,50,51]. For example, non-photosynthetic and photosynthetic heterokonts are distinct, while within apicomplexans, the relationships mirror those from ribosomal protein gene trees [44,52]. However, eIF4E-1 from the two syndineans (*Amoebophrya*) and *Perkinsus marinus* fall outside the apicomplexans and core dinoflagellate clade, while apicomplexans are embedded near the eIF4E-1c of core dinoflagellates. This branching pattern conflicts with both rDNA and ribosomal protein trees [44,53-55]. Additionally, within eIF4E-2, the ciliates form a clade next to heterokonts, and dinoflagellates with bootstrap support <60%, in contrast with the expected organismal relationships where ciliates would be more closely related to dinoflagellates. The large clades described below and named core dinoflagellate subclades were consistently recovered when different amino acid substitution matrices were used (Whelan and Goldman (WAG), Jones Taylor Thornton (JTT), and Le and Gascuel (LG) matrices), although there were some subtle changes in topology in areas of poor bootstrap support. When the JTT matrix was used the two *Chromera velia* sequences were placed together with apicomplexans, while in the LG tree (Figure 3) these sequences are on a very short branch outside of core dinoflagellate eIF4E-1c and apicomplexans. Also, the two *P. marinus* sequences in eIF4E-1 were placed outside of apicomplexans and core dinoflagellate eIF4E-1a,b and c under the JTT model (data not shown).

### Core dinoflagellate eIF4E-1 sub-clades

Core dinoflagellate eIF4E-1 contains four distinct monophyletic groups, each with at least one representative from each of the eleven core dinoflagellate species, which we have termed eIF4E-1a through -1d. Although bootstrap support for these groups was low when analyzed with the other two major eIF4E clades and outgroups, a re-analysis of eIF4E-1 containing only the core dinoflagellates produced a tree similar to that of the broader taxon sampling (Figure 3), but the sub-sampled tree had increased bootstrap support. Most notable was increased support for eIF4E-1d (100% from 36% bootstrap) and support for the combination of eIF4E-1a and -1b to 56%. Meanwhile, support remained high at 100% for eIF4E-1c.

There were different patterns of amino acid conservation between eIF4E-1a through -1d. eIF4E-1a was characterized by short branch lengths and high sequence identity. Selecting *Amphidinium carterae* and *Karlodinium veneficum* as representative core dinoflagellate species, there was 86% pairwise amino acid identity between the most closely related eIF4E-1a sequences across the entire sequence. This is roughly equal to the identity between the three eIF4E-1a copies in *K. veneficum* (83–87% identity). The eIF4E-1d is the next most conserved, with 70% identity between the two species. Divergence is higher in eIF4E-1b and -1c, with a pairwise identity between the same two species of 60% and 53%, respectively. We find asymmetric patterns of duplication across eIF4E-1a through -1d among core dinoflagellates. The rank order of number of copies per species was eIF4E-1a > −1d > −1b > −1c with eIF4E-1c having a single copy per species (Figure 3 inset). This is the same rank order that corresponds, from highest to lowest, to the pairwise similarity within each sub-clade.

### Core dinoflagellate eIF4E-2 sub-clades

Within core dinoflagellate clade 2 eIF4E, two sub-clades were found, eIF4E-2a and -2b. eIF4E-2a was found in all eleven species, with eIF4E-2b being found in only eight species. eIF4E-2a is more conserved than eIF4E-2b based on pairwise comparisons and branch lengths. In contrast to eIF4E-1, there is higher bootstrap support for both eIF4E-2a and -2b (69% for eIF4E-2a and 100% for eIF4E- 2b). There is a large amino acid extension at the carboxy terminus of eIF4E-2a ranging from 200 – 270 amino acids compared to eIF4E-2b, a region not used in the phylogenetic analysis. This region was difficult to align between species and contains simple amino acid repeats of one proline followed by two to four alanines.

In three apicomplexan genera, *Toxoplasma, Neospora* and *Plasmodium*, eIF4E sequences that segregate with clade 2 eIF4E were found in initial phylogenies and searches. These sequences are very divergent, with long-branch lengths. Candidate homologs are absent from other apicomplexan groups in BLAST searches when either *T. gondii* or *P. falciparum* were used as a query. After initial phylogenies, these sequences were excluded from phylogenetic trees due to long-branch lengths (the GenBank gi numbers are reported in Additional file 1).

**Figure 4:**
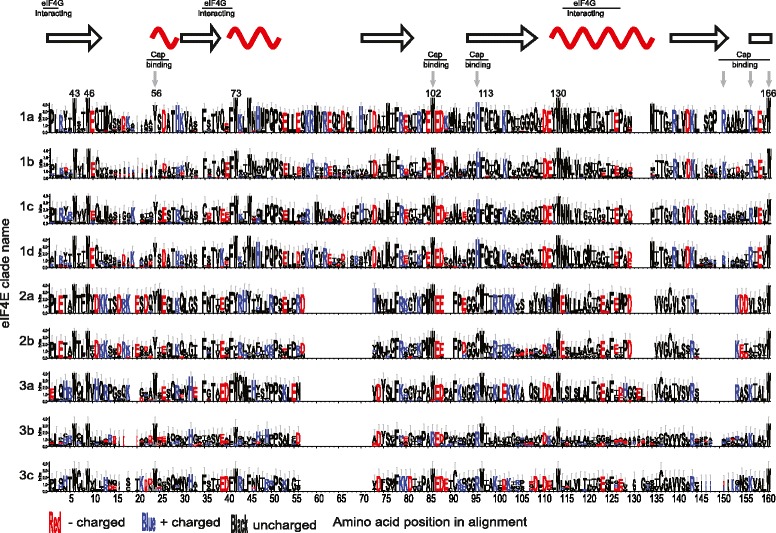
**Sequence logos for the core eIF4E domain for all nine core dinoflagellate sub-clades.** The alignment and gap arrangement are identical to that used for the phylogeny, although only core dinoflagellates are represented. The positions of the alpha helices and beta sheets of murine Class I eIF4E [PDB:1L8B] are shown using arrows to represent beta sheets and red curves for alpha helices. The cap binding residues and the eIF4G interacting domain are highlighted. The eight conserved aromatic residues are numbered according to the murine reference structure. Specific, conserved amino acids demonstrated to bind the mRNA cap in the murine model are highlighted with grey arrows. The legend for the amino acid coloring is displayed.

Heterokonts contain clades corresponding to both eIF4E-2 and metazoan Class II eIF4E (Figure 3) [33]. Heterokont Class II eIF4E form a distinct monophyletic group outside of alveolate eIF4E-2 and have the diagnostic metazoan Class II aromatic residue replacement at W43 to a tyrosine (Figures 3 and 4). Clade 2 eIF4E was not found in diatom genome datasets, but *Ectocarpus siliculosus*, *Albugo laibachii*, *Phytophthora infestans,* and *Phytopthora sojae* all contain both eIF4E-2 and a metazoan Class II eIF4E (Figure 3 and Additional file 3). Only *P. infestans* contains multiple members of eIF4E-2, so heterokont specific sub-clades could not be defined. These sequences were not given lettered sub-clade identification as the phylogeny did not demonstrate them to be homologous to the sub-clades found within the core dinoflagellates.

### Core dinoflagellate eIF4E-3 sub-clades

Only the core dinoflagellates and heterokonts contain clade 3 eIF4E, and homologous sequences were absent in the apicomplexan and ciliate genome data based on BLAST searches and phylogeny. eIF4E-3 from heterokonts form two clades corresponding to the photosynthetic and non-photosynthetic heterokonts. Only one eIF4E-3 was found for each heterokont species examined**.** In contrast, eIF4E sequences from the core dinoflagellates separate into three monophyletic groups within clade 3 to give eIF4E-3a, −3b, and -3c. Like eIF4E-1 and eIF4E-2a, eIF4E-3a contains sequences from all eleven core-dinoflagellate species and is more conserved than eIF4E-3b and -3c. eIF4E-3b and -3c are poorly represented with just six and five species, respectively. eIF4E-3b sequences have an extended amino-terminus compared to eIF4E-3a, that includes a DNA alkylation, or dioxygenase, domain, a divergent character not captured by the phylogeny of the aligned core region. Additionally, eIF4E-3b contained a number of sequences with amino acid compositional bias when compared with the remaining members of the gene family (Additional file 2).

### Comparison with eIF4E sequences from *Perkinsus marinus* and *Amoebophrya*

Within eIF4E-1, the two *Amoebophrya* strains, representing the syndineans each have two distinct copies, one forming an outgroup to the apicomplexan/core dinoflagellate clade, and a second copy nested near eIF4E-1a of core dinoflagellates. Neither of these placements is strongly supported by bootstrap values. For eIF4E-2, the syndineans have a single copy. The branching pattern and bootstrap support does not show a clear affinity with either eIF4E-2a or -2b, but based on sequence length, the *Amoebophrya* eIF4E-2 was more similar to eIF4E-2a. No eIF4E-3-like sequences were found in the genus *Amoebophyra*.

*Perkinsus marinus* falls within the dinoflagellate lineage based on molecular phylogenies, but outside of the core dinoflagellate and syndinean clades (Figure 2) [44,46,47,52]. The *P. marinus* eIF4E family contains a total of two eIF4E-1, one eIF4E-2 and four eIF4E-3 members. The eIF4E-1 and eIF4E-3 sequences form a single species-specific monophyletic group with bootstrap support of 92% and 90%, respectively. While the major clade affiliation was clear, the phylogeny did not suggest specific relationships between the *P. marinus* eIF4E and core dinoflagellate sub-clades.

### Broader phylogeny

The eIF4E phylogeny of apicomplexans, dinoflagellates and heterokonts was combined with the previously published analysis of Joshi *et al*. [33] and recapitulated the eight well-supported clades of that analysis. As was previously reported, the relationships between the eight clades is ambiguous with poor bootstrap support. Dinoflagellate, ciliate and apicomplexan (Alveolate) as well as heterokont eIF4E-1 were placed within the clade 8 of Joshi *et al*. [33] and the combined Alveolate and heterokont eIF4E-1 and −3 were recovered as monophyletic groups (Additional file 4). However, comparing the phylogeny in Figure 3 to the broader phylogeny, eIF4E-2 from heterokonts did not form a single clade and were placed outside of eIF4E-2 from dinoflagellates, albeit with poor support. Phylogenies that included the long-branch ciliates were even more difficult to interpret. The three major clades (eIF4E-1, −2, and −3) described here cannot be reliably matched to the three previously described eIF4E classes (Class I, Class II, and Class III) [33].

### Structural predictions across the three dinoflagellate eIF4E clades

The alignment of the core dinoflagellate eIF4E family members used to construct the phylogenetic tree contains insertions, deletions, and residue changes at important functional positions that differentiate the clades and sub-clades (Figure 4). The eight conserved aromatic residues of murine eIF4E have been mapped onto the alignment of dinoflagellate eIF4E sequences (Figure 4) as points of reference. The eIF4E-1 contains extended sequences between W73 and W102, and W130 to W166 compared to eIF4E-2 and −3. The function of the extension is not known, but the different eIF4E-1 sub-clades show marked heterogeneity at this region. In the core dinoflagellate clade 1 and clade 2 eIF4E, there is a tyrosine substitution at the position equivalent to W56. Conserved W102 is maintained across eIF4E sequences from all dinoflagellate clades except eIF4E-3b. In this case, the hydrophobic tryptophan has been substituted for an arginine. E103, which also interacts with the mRNA cap, is found at equivalent positions in all three dinoflagellate clades of eIF4E [31,32]. However, in clade 2 eIF4E, there is a small deletion C-terminal to it, as well as the introduction of a proline residue; an indicator of possible kinks in the polypeptide and an alteration of its function. The tryptophans at positions 43 and 46 are conserved, as is W166, which is involved in recognition of the methyl group on m^7^GTP.

In core dinoflagellate eIF4E-3 sequences, R112 is conserved, but there is a conservative substitution of histidine in eIF4E-1 and a cysteine substitution in eIF4E-2. In all three dinoflagellate clades, R157 may or may not be conserved, because the alignment of this residue is ambiguous near the insertion between W130 and W166 of dinoflagellate eIF4E-1 (Figure 4). eIF4E-3 contains the conserved K162 and in eIF4E-1 there is an arginine substitution, but this site is a hydrophobic valine or alanine in eIF4E-2.

In eIF4E family members capable of binding eIF4G, the consensus sequence of the recognition motif is S/TVxxFW ending at W73 [31,32,56-58]. In the core dinoflagellate eIF4E sequences, similar motifs are conserved. Although eIF4E-1 and −3 have a tryptophan at W73, eIF4E-2 has tyrosine, phenyalanine or leucine. In addition, there are subtle variations between the different eIF4E-1 sub-clades in the consensus sequence of the binding domain. The region can be polar (TVQeFW), as in eIF4E-1a and -1b, acidic (TVEEFW) as in eIF4E-1c, or basic (TVKgFW), as in eIF4E-1d. Overall, based on primary sequence alignment and structural predictions, the members of the core dinoflagellate eIF4E family are diverse at sites known to be functionally important in mouse and other model organisms. Hence, functional analysis will be necessary to determine the roles of different family members.

## Discussion

### Evolutionary patterns in the eIF4E family

The combination of relatively dense taxon sampling and known relationships has allowed us to make clear comparisons of the eIF4E genealogy to the organismal relationships, revealing both the relative timing and degree of duplication within core dinoflagellates. Prior to diversification of heterokonts and alveolates the eIF4E gene family contained at least three clades. These clades were retained in heterokonts and dinoflagellates, but apicomplexans and ciliates have apparently lost eIF4E-2 and −3 (Figure 3). Other shared eIF4E family members may have been lost from either dinoflagellates or heterokonts or both. The large sequence divergence between the three clades implies different functions, so that the common ancestor is likely to have used the gene family in three different roles. However, in the core dinoflagellates the gene family has expanded mostly via relatively recent, lineage specific duplications, into nine distinct eIF4E sub-clades. Additional duplications within sub-clades have been found, so that some species have multiple subtly different copies. The multiple eIF4E family members have the potential to create a customized toolkit for regulating gene expression as in plants and metazoans. In contrast, heterokonts have only four and apicomplexans and ciliates only one or two eIF4E family members, suggesting that different lineages have placed different reliance on, and approaches to, translational control of gene expression.

### Matching eIF4E structural classes to clades

Based on phylogenetic analysis the three eIF4E clades described here cannot be mapped to the three previously defined eIF4E structural classes in animals and plants (Additional file 4) [30,33]. The broad eIF4E phylogeny is poorly resolved and the underlying organismal relationships between metazoa, plants, heterokonts and alveolates are uncertain (Additional file 4) [30,33]. For example, based on the phylogeny, the dinoflagellate eIF4E-1 defined here has no obvious candidate ortholog in plants or metazoans. The eIF4E-1 sequences from alveolates and heterokonts contain an amino acid insertion (Figure 3) between W73 and W102 (numbering equivalent to murine eIF4E), a feature not seen in any plant or metazoan eIF4E family member (Figure 4). Alternately, plant and metazoan Class II eIF4E family members are defined in part by a tyrosine, phenylalanine or leucine at the position equivalent to murine W43. By this definition, no Class II eIF4E sequences have been found in dinoflagellates or ciliates. However, heterokonts do contain eIF4E family members defined as metazoan Class II based on this diagnostic character. The non-reciprocal mapping of clades to classes suggests diverse approaches to eIF4E functions in eukaryotes with loss and duplication occurring multiple times independently within plants, fungi, metazoans, alveolates and heterokonts.

### Different functions are likely among the dinoflagellate eIF4E family members

For the few gene families that have been studied in core dinoflagellates, a general pattern of highly duplicated but conserved genes in tandem arrays has been seen for actin, proliferating cell nuclear antigen, and the peridinin chlorophyll protein [9,11,12,14,15]. The different sequences typically have few amino acid differences and many synonymous nucleotide substitutions, suggesting little functional difference between the resultant proteins. Several other gene families, such as RuBsiCO, luciferase, and the light harvesting complex protein are polyproteins, with tandemly repeated protein units encoded on a single mRNA [10,13,59]. Our work shows that the eIF4E family contrasts with these other examples because the divergences are seen at critical amino acids suggesting the family members are functionally distinct. The broad species representation we used has allowed inferences about the relative timing of duplication and species divergence to be made and suggests that the different sub-clades within eIF4E-1 were likely present in the common ancestor of core dinoflagellates. Differences in duplication and divergence within each clade and sub-clade may reflect a form of genomic dose effect, such that the more essential eIF4E forms are also the more duplicated and possibly more specialized. Thus, although the functionalities present in the common ancestor of alveolates and heterokonts are likely to be retained in the three clades, the sub-clades present in the core dinoflagellates are likely to play distinct roles in the cell and correlate with an increased reliance on post-transcriptional control.

### Dinoflagellate clade 1 eIF4E is likely to contain at least one translational initiation factor

Of the three major clades in the phylogeny shown here (Figure 3), eIF4E-1 was the only clade that included sequences from every species surveyed. Within core dinoflagellates, eIF4E-1 stands out as the most duplicated, ranging from 5 to 9 copies per species and is also the most conserved. Within eIF4E-1a, only 15% of amino acid sites were found to be variable across the entire coding sequence, and the changes represent functionally conservative substitutions, *i.e.* high similarity or many positives in pairwise comparisons. Although all plant, fungal, and metazoan translation initiation factors are members of Class I, not all Class I eIF4E members are initiation factors [34,60]. By analogy, not all dinoflagellate clade 1 eIF4E family members may be functional translational initiation factors. Based on conservation and duplication, we predict that the primary workhorse translation initiation factor will be found within eIF4E-1, as this appears to be the only eIF4E present in apicomplexans. Also, if the theory of dose compensation holds true, the rank order of number of copies per species; *i.e.* eIF4E-1a > d > b > c, would suggest that eIF4E-1a functions as the primary translation factor.

In looking for different roles of the other sub-clades in the regulation of gene expression, a variety of possibilities exist. Simplistically, should all the eIF4E-1 function as translation initiation factors, eIF4E-1a could be involved in recruitment of the most commonly translated mRNAs, followed by -1d, −1b, and -1c. Examples of this can be seen in certain metazoan Class I eIF4Es. For instance, in *Caenorhabditis elegans*, functional differences are found within the different members of Class I eIF4E. At specific developmental stages, *C. elegans trans*-splices sub-sets of mRNAs with a spliced leader containing a trimethyl cap structure [61]. In *C. elegans*, m^7^GTP and trimethyl mRNA caps are bound by different Class I eIF4E family members, IFE3 versus IFE1, 2 and 5, respectively [62-64]. Although all core dinoflagellate mRNAs are presumed to be trans-spliced, it seems that the first nucleotide after the methylated base can be variable, opening up the possibility that the four eIF4E-1 sub-clades could recruit mRNAs with different cap structures [65-67].

The conservation found in clade 1 eIF4E does not extend to the eIF4G binding domain, in which there are amino acid substitutions within each sub-clade that are likely to have strong affinities for different binding partners. The eIF4G binding domain is also the site of interaction of a range of eIF4E binding proteins that use the same YXXXXLφ sequence to competitively bind eIF4E, preventing eIF4E–eIF4G interactions and inhibiting cap-dependent translation and/or targeting mRNAs to specific locations [34,60]. The variable sequence of the eIF4G binding domain represents a strategic point for selection of mRNAs. Different eIF4E and eIF4G combinations could favor the translation of different subsets of mRNA thus providing a toolkit for regulation of gene expression post-transcriptionally as has been proposed in *Drosophila melanogaster* [41,68] and *Leishmania* [69,70].

### Dinoflagellate eIF4E-2 has an alternate function in the toolkit

Core dinoflagellate eIF4E-2 have substitutions at two positively charged residues known to be involved in cap binding, R112 and K162 (Figures 1 and 4). In eIF4E-1, a histidine is conserved in all sub-clades (Figure 4), but in eIF4E-2, cysteine is substituted at position 112. In eIF4E-1 at K162 a conserved arginine is present in all sub-clades, but this has been substituted by a valine in eIF4E-2. These substitutions could inhibit interaction with the negatively charged phosphate groups linking the cap to the mRNA. Furthermore, there is a two amino acid deletion near the conserved W102 involved in cap binding. In consideration of these differences, we predict that eIF4E-2 does not function well as a cap binding protein *in vitro*.

Differences in cap binding ability are reminiscent of the metazoan Class II and Class III eIF4Es that cannot compete *in vitro* with Class I eIF4E for mRNA and are not found associated with eIF4G in cells [38,39,43,71]. However, the affinity of metazoan Class II eIF4E, to bind mRNA 5′-caps is increased by interaction with a variety of partner proteins that, along with metazoan Class II eIF4E function to repress specific mRNA translation [38,39,43,71]. Similarly, metazoan Class III eIF4E specifically binds the m^7^G cap in the absence of an aromatic sandwich, using instead different spatial arrangements of residues to provide the necessary electrostatic and van der Waals contacts [43,71]. Although the eIF4E from Class III has a lower affinity than Class I eIF4E for mRNA caps *in vitro*, in cells nearly all of the endogenous mouse Class III eIF4E is in the cap-bound fraction suggesting that factors in the cell increase the affinity of Class III eIF4E for the cap [43,71]. Given these examples in metazoa, and the key substitutions in eIF4E-2 cap binding residues, dinoflagellate eIF4E-2 alone are unlikely to bind cap, but may bind in the presence of interacting partner proteins.

Clade 2 eIF4E is also unique in that the degree of duplication is roughly comparable within core dinoflagellates and heterokonts. Heterokonts contain two different cognates within eIF4E-2, but only one was allied with the dinoflagellates, while the other is allied with Class II eIF4E from metazoans and plants. Whether or not the placement of heterokont sequences with Class II sequences is convergent evolution cannot be stated from these data. However, retention of eIF4E-2 in heterokonts and dinoflagellates (both core dinoflagellates and syndineans) implies a conserved function that does not rely on traditional cap binding. Also the extension at the carboxy terminus of eIF4E-2b indicates eIF4E-2b and possibly eIF4E-2 interact with binding partners that are not associated with eIF4E-1 or −3. The absence of eIF4E-2 in several of the alveolate lineages and differences in duplication when comparing dinoflagellates and heterokonts makes it likely that eIF4E-2 performs a non-essential regulatory role which has become more specialized within the dinoflagellates.

### Core dinoflagellate eIF4E-3 have many attributes in common with the metazoan Class I eIF4E

Unlike core dinoflagellate eIF4E-1 and eIF4E-2, eIF4E-3a and -3c have a tryptophan at position 56 and show complete conservation of the amino acids important in charge neutralization of the phosphate bridge of the mRNA cap. They do not have the insertions uniquely characteristic of eIF4E-1. In addition, the eIF4G interacting motif more closely resembles that of the metazoan Class I eIF4E. However, for multiple considerations, eIF4E-3 has not been considered a good candidate for the workhorse translation factor. These include the fact that eIF4E-3 is found only in the core dinoflagellates and heterokonts, and is absent in the apicomplexan and ciliate genome data. Additionally, only one eIF4E-3 is found for any heterokont species examined with the two clades corresponding to the photosynthetic and non-photosynthetic heterokonts. Furthermore, only one eIF4E-3 sub-clade, eIF4E-3a, is represented in all eleven core-dinoflagellate species with eIF4E-3b and eIF4E-3c represented in just six and five species, respectively. Moreover, the fusion of the alkylation or dioxygenase domain on the N-terminus of eIF4E-3b is remarkable and unprecedented, making hypotheses about its function unrealistic without further experiments. This does not mean, however, that eIF4E-3 does not contain a translation factor.

There are certainly differences in the conserved cap binding pocket between members of eIF4E-1 and −3 and it is not clear that individual spliced leader mRNA in dinoflagellates possess an identical cap [65-67]. Differences in mRNA caps could allow specific translation of genes involved in stress response or other unique pathways that would not be constitutively expressed. Conversely, expression of members of eIF4E-3 could be situational; only occurring during specific developmental stages and not detectable at other times.

## Conclusions

Dinoflagellate genomes contain an eIF4E family that demonstrates a greater diversity and degree of duplication than has been seen in other eukaryotes [30]. The general model for metazoan eIF4E family members is to have different cellular roles related to post-transcriptional regulation of gene expression. These different roles are supported by phylogeny and specific amino acid changes mapped onto the protein structure [33]. Likewise, dinoflagellate eIF4E family members are expected to have distinct functions based on this phylogeny, with their unique diversity allowing for an increased dependence on the translational control of gene expression. Only through structural and functional studies can we confirm these predictions made from the phylogeny.

## Methods

### Taxon sampling

Data from Illumina RNA-seq libraries were used for eleven core dinoflagellate species and two syndineans. Six of these, *Akashiwo sanguinea, Amphidinium carterae, Gyrodinium instriatum, Karlodinium veneficum, Polarella glacialis,* and *Prorocentrum minimum* were sequenced with 100 base paired-end reads with 30 to 100 million reads collected per species and assembled with Trinity [44,72] (Additional file 1). The two syndineans, *Amoebophrya* parasites specific for *Akashiwo sanguinea* and *Karlodinium veneficum,* respectively, were co-cultured and sequenced with their hosts as previously described [73]. Parasite sequences were deconvoluted from host using either using AT bias for the parasite from *Karlodinium veneficum*, or by phylogenetic analysis for *Amoebophrya* sp. ex *Akashiwo sanguinea* as done previously [74]. In addition, Moore Foundation Illumina datasets were downloaded for five core dinoflagellates. These consisted of 50 base paired-end reads for *Alexandrium tamarense, Karenia brevis, Lingulodinium polyedrum,* and *Scrippsiella trochoidea*. The sequences were downloaded from the Community cyberinfrastructure for Advanced Microbial Ecology Research and Analysis, CAMERA, database (http://camera.crbs.ucsd.edu/mmetsp/) and assembled using CLC Genomics (Qiagen, Boston, MA). Transcriptome data for *Symbiodinium* sp. B1 were downloaded from http://marinegenomics.oist.jp/genomes/downloads?project_id=21 [7].

In addition to the eleven core dinoflagellates and two syndinians, the genome sequences from *Perkinsus marinus* and twelve apicomplexans [see Additional file 1] were also used. Data from GenBank’s reference sequence (ref_seq) protein or non-redundant nucleotide database were downloaded and formatted into blast databases for each species. In total, sequences from forty-nine different species were used in this study [see Additional file 1].

### eIF4E sequence retrieval and annotation

The blastx or blastp programs were used with an e-value cut off of 1E^−10^ and a suite of eIF4E query sequences [30]. For dinoflagellates, the set of eIF4E sequences from two representative species, *Amphidinium carterae* and *Karlodinium veneficum* were used as queries. For heterokonts, eIF4E sequences from *Thalassiosira pseudonana* and *Phaeodactylium pseudonana* were used as queries. For apicomplexans, *Toxoplasma gondii* and *Plasmodium vivax* were used as queries. Such searches consistently returned pairwise alignments of >130 aligned amino acids. The resulting eIF4E sequences were aligned using the Clustal Omega program with the full iteration option [75].

Sequences from the representative species within each clade identified in the phylogeny were used as queries in subsequent blast searches to improve representation. This iterative process was repeated until no novel eIF4E family members were found. For a few sequences, manual assembly of scaffolded sequences was required to obtain complete coding regions. In such cases, promising blast hits (e-values < 1E^−10^) with short alignments were examined using read mapping to identify and then manually assemble fragmented gene copies. This was done using Bowtie2 read mapping of reads to contigs, followed by visualization using the Integrative Genomics Viewer (IGV) to identify read mates, and finally by assembly using short overlap consensus in Sequencher (Gene Codes, Ann Arbor, MI) [76,77]. A putative heterokont contaminant was found in the sequence data from *Alexandrium tamarense* and removed from further analyses (Additional file 4).

The alignments were trimmed to the eIF4E core region from five amino acids upstream of W43 to ten amino acids downstream of W166 numbered by murine eIF4E [PDB:1L8B] sequence positions [33]. The sequence alignment was submitted to TreeBase with accession number [S16938]. Phylogenetic analysis was performed using RAxML with 1000 bootstrap replicates. The LG amino acid substitution matrix with gamma rate correction was selected based on hierarchical likelihood ratio tests of the optimal tree calculated using the JTT amino acid substitution matrix (the PROTGAMMAJTT model in RAxML) [78]. Using the amino acid frequencies from the LG matrix resulted in better likelihood scores than the empirical amino acid frequencies in the alignment, and similar results were seen with the WAG and JTT matrices. All the analyses assumed a single stationary model across the tree. An additional phylogeny was constructed using the supplemental data from Joshi *et al*. and adding in the sequences from the present study with 100 bootstrap replicates, although in this case the JTT model was used [33]. For this combined analysis, the ciliates were excluded due to long-branch lengths.

Amino acid bias was calculated using Tree-Puzzle which uses a chi-squared test to compare the amino acid composition of each alignment row with the empirical frequencies calculated from the alignment [79]. The amino acid bias for specific clades was further explored using the composition profiler tool [80].

Sequence logos were created using WebLogo [81] for each core dinoflagellate clade by trimming outgroup taxa and creating clade specific alignments. The amino acid sequences were aligned, and the secondary and tertiary structures predicted using an online Protein Homology/analogY Recognition Engine (PHYRE) [82], with [PDB:1L8B] as a reference. The resulting PDB file was then displayed with Visual Molecular Dynamics software (http://www.ks.uiuc.edu/Research/vmd/) [83].

### Availability of supporting data

Alignments used in this study were deposited in TreeBase with accession # *S16938*, see http://purl.org/phylo/treebase/phylows/study/TB2:S16938.

## Additional files

Additional file 1:
**Supplemental table with gi numbers, bioproject numbers and other publicly available data sources.**
Additional file 2:
**Amino acid compositional bias plots for specific clades with amino acid bias.**
Additional file 3:
**A nexus format tree of Figure** 2 **with alignment.**
Additional file 4:
**A more broadly sampled eIF4E phylogeny.**
