# Supplementary material for: The alveolate translation initiation factor 4E family reveals a custom toolkit for translational control in core dinoflagellates
Source: BMC Evol Biol. 2015 Feb 10;15(1):14. doi: 10.1186/s12862-015-0301-9 (PMC4330643; doi:10.1186/s12862-015-0301-9)

eIF4E-3b from core dinoflagellates versus remaining unbiased sequences in alignment

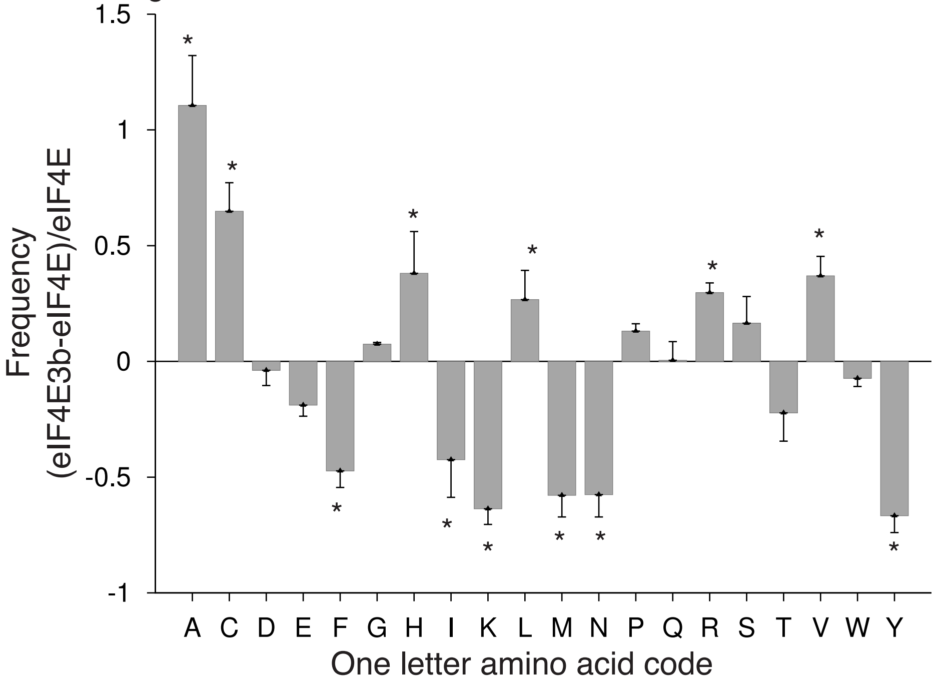

Ciliate eIF4E sequences versus remaining unbiased sequences in alignment

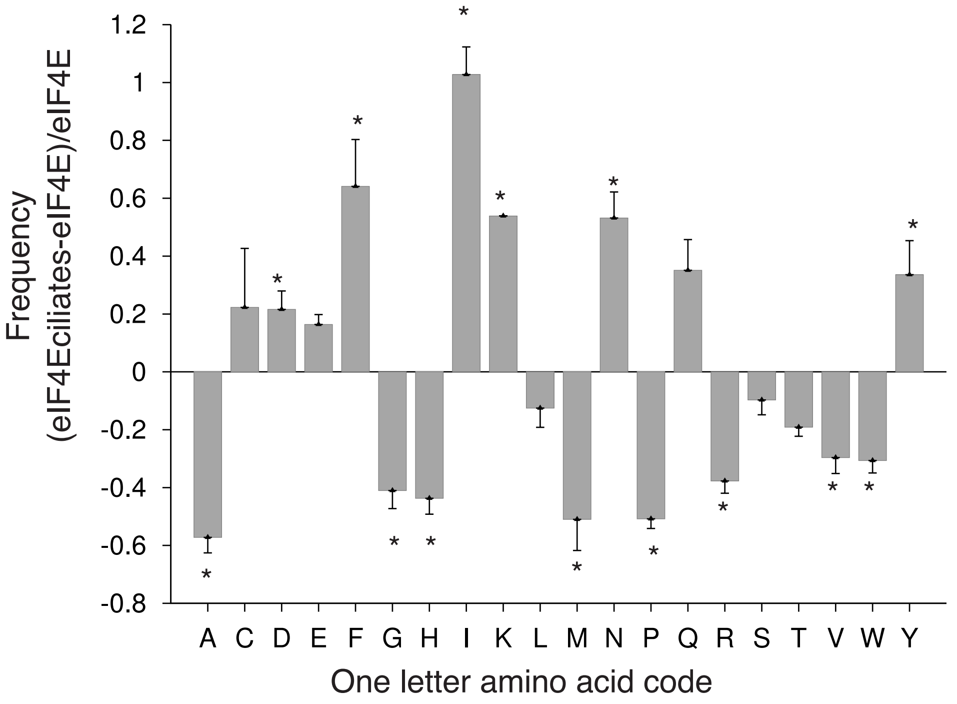

Supplement: Additional file 2: — Amino acid compositional bias plots for specific clades with amino acid bias. [file 12862_2015_301_MOESM2_ESM.pdf]
