# Supplementary material for: The alveolate translation initiation factor 4E family reveals a custom toolkit for translational control in core dinoflagellates
Source: BMC Evol Biol. 2015 Feb 10;15(1):14. doi: 10.1186/s12862-015-0301-9 (PMC4330643; doi:10.1186/s12862-015-0301-9)
Supplement: Additional file 4: — A more broadly sampled eIF4E phylogeny. [file 12862_2015_301_MOESM4_ESM.pdf]

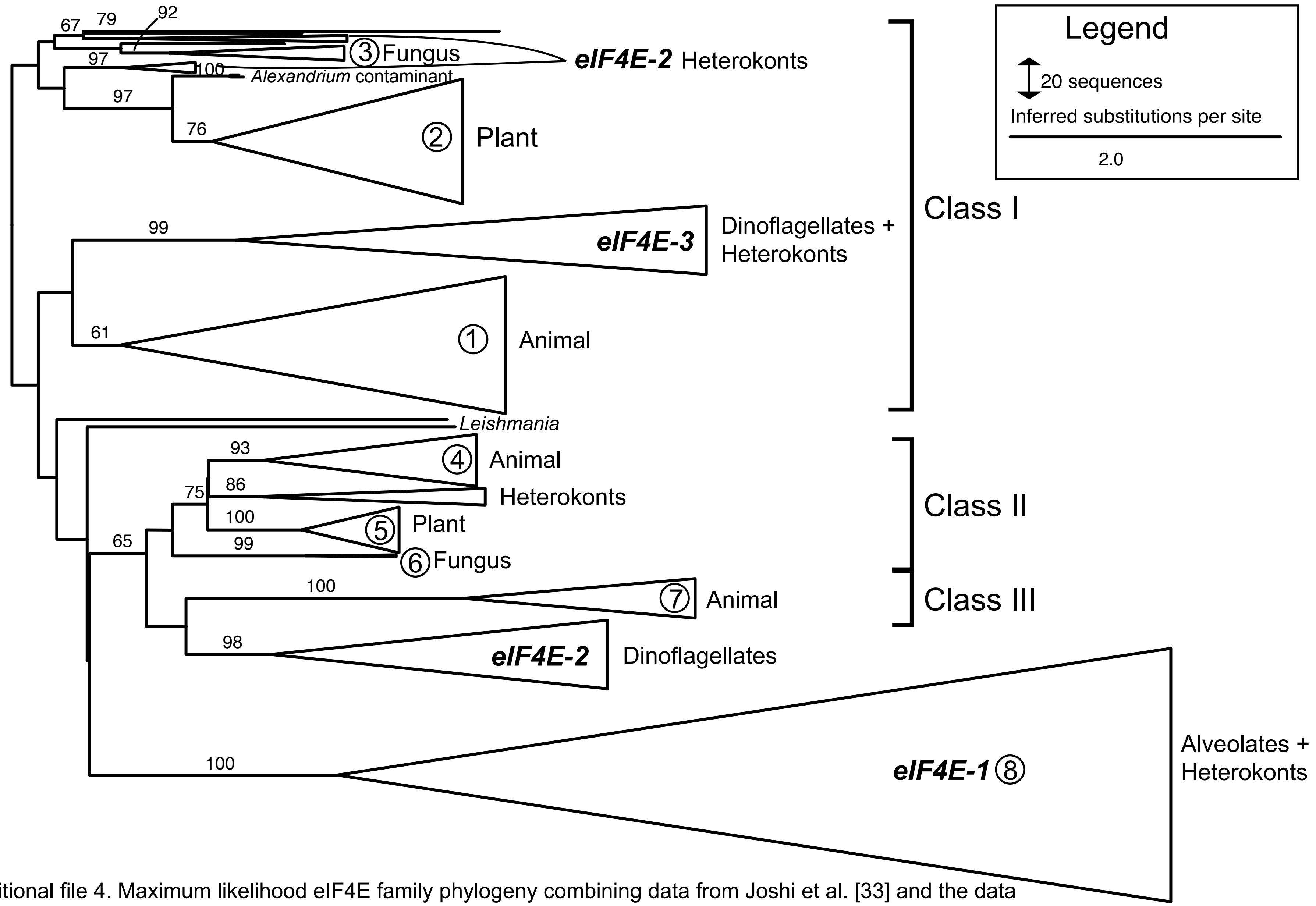

Additional file 4. Maximum likelihood eIF4E family phylogeny combining data from Joshi et al. [33] and the data used to generate Figure 2. The JTT with gamma site to site rate variation model and 100 bootstrap replicates were used. The eight clade names from Joshi et al. [33] are shown as circled Arabic numerals inside or beside the appropriate clades with the class designation shown outside the brackets. The clade designations from Figure 2 are shown in italics. Bootstrap support values (100 replicates) greater than 60 % are shown above branches. The horizontal scale is inferred substitutions per site and the vertical scale is sequences per clade.
